# Pentose Phosphate Pathway Regulates Tolerogenic Apoptotic Cell Clearance and Immune Tolerance

**DOI:** 10.3389/fimmu.2021.797091

**Published:** 2022-01-10

**Authors:** Dan He, Qiangdongzi Mao, Jialin Jia, Zhiyu Wang, Yu Liu, Tingting Liu, Bangwei Luo, Zhiren Zhang

**Affiliations:** ^1^ Medical College of Chongqing University, Chongqing, China; ^2^ Research Center for Integrative Medicine of Guangzhou University of Chinese Medicine, Guangzhou, China; ^3^ Institute of Immunology, Army Medical University, Chongqing, China

**Keywords:** pentose phosphate pathway (PPP), macrophage, efferocytosis, immune tolerance, autoimmune disease

## Abstract

The efficient removal of apoptotic cells (ACs), a process termed as efferocytosis, is essential for immune homeostasis. While recent work has established an important interplay between efferocytosis and cellular metabolic changing, underlying mechanisms remain poorly known. Here, we discovered that pentose phosphate pathway (PPP) regulates tolerogenic ACs clearance and immune tolerance. ACs decreased levels of PPP-related genes and metabolites in macrophages. AG1, the agonist of PPP, increased the activity of PPP but greatly reduced macrophage phagocytosis of ACs and enhanced the inflammatory response during efferocytosis. miR-323-5p regulated the expression of PPP-related genes and its levels increased during efferocytosis. miR-323-5p inhibitor greatly promoted levels of PPP-related genes, reduced the macrophage phagocytosis of ACs, and increased inflammatory response during efferocytosis, suggesting that miR-323-5p was essential in regulating PPP activity and ACs clearance in macrophages. Correspondingly, the PPP agonist AG1 exacerbated the lupus-like symptoms in the AC-induced systemic lupus erythematosus (SLE) model. Our study reveals that regulating PPP-dependent metabolic reprogramming is critical for tolerogenic ACs phagocytosis and immune tolerance.

## Introduction

In our body, billions of cells die every day as part of normal homeostasis. Thereafter, ACs are quickly recognized and cleared by phagocytes. The clearance of ACs activates immunosuppressive pathways and promotes the production of anti-inflammatory cytokines to remain immunological hemostasis. Consequently, when the engulfment system does not function well, uncleared ACs can undergo secondary necrosis, losing membrane integrity and releasing noxious intracellular molecules into the surrounding tissue, which plays an important role in inducing autoimmune diseases such as systemic lupus erythematosus (SLE), rheumatoid arthritis, and glomerulonephritis. Thus, the efficient clearance of ACs is essential for maintaining tissue homeostasis and immune tolerance in multicellular organisms (1–3).

Phagocytic clearance of ACs involves several events that ensure immunologically silent elimination of dying cells. AC removal is believed to be triggered by the AC-released “find me” signals that recruit phagocytes. ACs expose the plasma membrane inner leaflet phospholipid phosphatidylserine or its oxidized forms to serve as “eat me” signals to distinguish them from viable cells. Phagocytes interact with ACs *via* various engulfment receptors, directly binding with eat-me signals on ACs, or indirectly recognizing bridging molecules that bind to eat-me signals. Following AC engulfment, phagocytes suppress the production of pro-inflammatory cytokines and increase the release of anti-inflammatory cytokines to prevent immune responses against self-antigens (1–4). However, little is known about how dying cells affect phagocyte signaling pathways related with the engulfment of ACs and the subsequent activation of tolerogenic pathways.

It is now well appreciated that specific cellular metabolic changes are closely related to immune cell functions (5). In the case of efferocytosis, early investigations indicate that during efferocytosis, AC-derived fatty acids and sterols activate endogenous receptors such as PPARδ (6) and LXR (7), further increasing efferocytosis and enhancing anti-inflammatory response in macrophages. Recently, Zhang et al. also showed that efferocytosis significantly enhanced fatty acid oxidation and activated the respiratory chain to induce the expression of IL-10 (8). In parallel, Morioka et al. discovered that phagocyte glycolysis contributed to the continued engulfment of ACs and lactate released *via* SLC16A1 promoted anti-inflammatory response in the early stages of efferocytosis (9). Meanwhile, mitochondrial uncoupling protein 2 (10) and mitochondrial fission (11) promote the continued clearance of dying cells by phagocytes. These observations highlight the important interplay between efferocytosis and cellular metabolic changes, which may provide exciting new avenues for harnessing impaired efferocytosis and related diseases.

The pentose phosphate pathway (PPP), which is a way of oxidative decomposition of glucose, starts with glucose 6-phosphate (G-6-P) and finally produces NADPH and ribose-5-phosphate. G6PDH and 6PGDH that catalyze the two-step irreversible dehydrogenation reactions in this process are the rate-limiting enzymes of PPP. It is appreciated that PPP is related to macrophage polarization and function (12, 13). M1 macrophages show increased PPP activity and M2 macrophages show decreased PPP activity. Moreover, different activity of the PPP regulates the functional diversity of macrophages (14). While our previous study showed that Dicer promoted the AC clearance through PPP (15), contributions of PPP to AC clearance and immune tolerance remain unknown. Here, we found that PPP regulated tolerogenic AC clearance and immune tolerance.

## Materials and Methods

### Animals

All mice were raised under pathogen-free conditions in the animal facility of Army Medical University. The animal study was reviewed and approved by the local Administration District Official Committee of Army Medical University, Chongqing, China. The C57BL/6J mice were purchased from Byrness Weil Biotech Ltd, Chongqing, China. For SLE model induction (16), 8-week-old female mice were used. A total of 1.5 × 10^7^ apoptotic thymocytes suspended in sterile phosphate buffer were injected intravenously into anesthetized mice once a week for four weeks; after 15 days of rest, the injections were repeated twice, and the mice were euthanized after 1 month for SLE evaluation. Meanwhile, 24 h before apoptotic cell injection, AG1 (10 mg/kg, i.p.) was administered weekly (AG1 is still injected at a fixed time during the 15-day break). After the last apoptotic cell injection, AG1 was injected twice per week. The same volume of PBS was injected into the control group.

### Generation of Apoptotic Cells

Thymocytes were obtained from the thymus of 4- to 6-week-old female C57BL/6 mice by grinding with a 70-μm cell strainer. Red blood cells were lysed with red blood cell lysis buffer (TIANGEN, Beijing, China). Thymocytes were washed twice in PBS and treated with 1 μmol/L dexamethasone (Sigma-Aldrich Corp, Darmstadt, Germany) for 4–6 h at 37°C in RPMI supplemented with 10% fetal bovine serum (FBS, Gibco BRL, Grand Island, NY, USA) to generate apoptosis. Jurkat cells were ultraviolet radiated for 15 min and incubated for another 4 h at 37°C in RPMI with 10% FBS to induce apoptosis. Cells were collected by centrifugation at 1,000 rpm for 5 min, washed three times in PBS, then resuspended in PBS or corresponding medium to prepare for use.

### *In Vitro* Phagocytosis Assay

Peritoneal macrophages were obtained by intraperitoneal injection of 3% Brewer’s thioglycolate (6) (Sigma-Aldrich Corp, Darmstadt, Germany) into mice for 72 h. Peritoneal lavage fluid was collected with 5 ml of precooled PBS. Primary peritoneal macrophages were washed twice after lysis of red blood cells, resuspended in medium, and then plated in 6-well plates in DMEM with 10% FBS. We discarded non-adherent cells after 4 h. They were pre-treated with AG1 (3 μM, MedChemExpress, Monmouth Junction, NJ, USA) or PBS (control) for 24 h. Apoptotic cells were labeled with pHrodo™ Green (Thermo Fisher Scientific, Carlsbad, CA, USA), a pH-sensitive phagocytosis-dependent indicator, for 10 min at 37°C, then pHrodo-labeled apoptotic cells were added at a ratio of 1:5 (macrophages:apoptosis cells) at 37°C in DMEM with 10% FBS. After 60 min, macrophages were centrifuged at 1,000 rpm for 5 min, washed twice, resuspended in PBS, and stained with APC anti-mouse F4/80 Antibody (BM8, BioLegend, San Diego, USA); they were later analyzed by flow cytometry.

### *In Vivo* Phagocytosis Assay

Mice were pre-treated with AG1 (10 mg/kg, i.p., MedChemExpress, Monmouth Junction, NJ, USA) or PBS (control) for 24 h before apoptotic cell injection. For *in vivo* peritoneal macrophage phagocytosis assays, 10- to 12-week-old mice were injected intraperitoneally with 2 × 10^7^ pHrodo-labeled apoptotic thymocytes 3 days after induction of peritonitis by thioglycolate. The mice were sacrificed an hour later and peritoneal lavage was collected with 5 ml of PBS. Cells were washed twice after lysis of red blood cells, resuspended in PBS, blocked with Fc receptors by incubating with anti-CD16/32 antibodies, and then were stained with APC anti-mouse F4/80 Antibody (BM8, BioLegend, San Diego, USA) to identify macrophages. Cells were analyzed by flow cytometry to quantify the number of peritoneal macrophages phagocytosing apoptotic thymocytes.

For *in vivo* splenic macrophage uptake assays, 8 × 10^7^ pHrodo-labeled apoptotic thymocytes were intravenously injected into 10- to 12-week-old female mice; 2 h after apoptotic thymocyte injection, the mice were sacrificed and their spleens were collected. Splenocytes were obtained by grinding with a 70-μm cell strainer. Single splenocyte suspensions were handled like peritoneal lavage fluid, and then cells were analyzed by flow cytometry to determine the number of spleen macrophages ingesting pHrodo-labeled apoptotic thymocytes.

### Flow Cytometry

Single-cell suspensions that we collected were washed twice, resuspended in staining buffer, and incubated with anti-CD16/32 antibodies (Sungene Biotech, Tianjin, China) for 15 min to block Fc receptors (0.5 μg/million cells). Then, the cell surface markers were stained by corresponding labeled antibodies diluted in staining buffer (0.2 µg/million cells in 100 µl volume) for 15 min at 4°C. Following staining, cells were washed and suspended in PBS and then analyzed immediately on CANTO II (Becton Dickinson, Sunnyvale, CA, USA). Flow data were collected with CellQuest Software and analyzed with FlowJo software.

### Prediction of miRNAs Target

The miRNAs targets were determined using two independent databases. The mirwalk (http://mirwalk.umm.uni-heidelberg.de/) was first used to search for miRNAs regarding pentose phosphate pathway (PPP), and 9,424 miRNAs were obtained. Afterwards, based on the core enrichment gene, G6pdx, Pgd, and H6pd were obtained by GSEA enrichment, using cytoscape merged to get 13 predicted miRNAs. Subsequently, they were sorted with TargetScan score (http://www.targetscan.org/).

### Metabolomics Analysis

Peritoneal macrophages from WT mice were cultured at 37°C in DMEM supplemented with 10% FBS. Peritoneal macrophages were incubated with apoptotic human Jurkat T cells at a ratio of 1:5 (macrophages:apoptosis cells) or control for 6 h. After washing, cells were cultured for another 4 h and then collected. Metabolite analysis was performed by liquid chromatography–tandem mass spectrometry. Samples were frozen and thawed 3 times with liquid nitrogen, and then sonicated in an ice water bath for 10 min to extract metabolites. After measuring the protein concentration, samples were added with acetonitrile and methanol (at 1:1 ratio), vortexed for 30 s, incubated at −40°C for 1 h, and then centrifuged at 12,000 rpm for 15 min at 4°C. The supernatant was transferred into an EP tube and dried under vacuum. The mixture of acetonitrile:methanol:water = 2:2:1 and isotopically labeled internal standard was then added in proportion. After 30-s vortex, the samples were sonicated in an ice-water bath for 10 min. The samples were then centrifuged at 12,000 rpm for 15 min at 4°C. The supernatant was transferred to a fresh glass bottle for analysis. Quality control (QC) samples were made by mixing aliquots of the supernatant from all samples. LC-MS/MS analyses were carried out using a UHPLC system (Vanquish, Thermo Fisher Scientific, Waltham, MA, USA) with a UPLC BEH Amide column (2.1 mm × 100 mm, 1.7 μM) coupled to a Q Exactive HFX mass spectrometer (Orbitrap MS, Thermo Fisher Scientific, Waltham, MA, USA). The A phase of the liquid chromatography was aqueous containing 25 mmol/L ammonium acetate and 25 mmol/L ammonia, and the B phase was acetonitrile. The autosampler temperature was 4°C and the injection volume was 3 μl. The raw data were processed using ProteoWizard software (17). Peak identification, peak extraction, peak alignment, and integration were performed using the R package written by Biotree (kernel XCMS), and then matched with the BiotreeDB (V2.1) self-built secondary mass spectrometry database for substance annotation (18).

### ELISA and Biochemical Parameters

NADPH and reduced glutathione were examined using an NADP+/NADPH Assay Kit with WST-8 (Beyotime, Jiangsu, China) and GSH and GSSG Assay Kit (Beyotime, Jiangsu, China) according to the manufacturer’s protocol. The prominent marks of SLE, anti-dsDNA antibodies (ADA), and anti-nuclear antibodies (ANA) in serum were measured respectively using Mouse Anti-dsDNA IgG ELISA Kit (Alpha diagnostics, Santa Monica, CA, USA) and Mouse ANA/ENA IgG ELISA Kit (Alpha diagnostics, Santa Monica, CA, USA), according to the manufacturer’s instructions.

### Real-Time Quantitative PCR

RNA Extraction from Cells was performed by using the RNAfast200 Reagent (Fastagen, Shanghai, China). Reverse Transcription used the cDNA reverse transcription kit (Takara, Shiga, Japan). Real-time PCR used SYBR Green qPCR Master Mix (MedChemExpress, Monmouth Junction, NJ, USA). The mRNA levels were normalized to β-actin as an internal control. The gene expression values were presented as the relative mRNA level (fold change) compared to untreated controls, whose expression levels were set as 1. The primers (Invitrogen, Carlsbad, CA, USA) used to measure the gene expression are listed as follows: β-actin (forward, 5′-TGGAATCCTGTGGCAT CCATGAAA-3′; reverse, 5′-TAAAACGCAGCTCAGTAACAG TCCG-3′), IL-1β (forward, 5′-GAAATGCCACCTTTTGACAG TG-3′; reverse, 5′-TGGATGCTCTCATCAGGACAG-3′), IL-6 (forward, 5′-TGATGGATGCTACCAAACTGG-3′; reverse, 5′-T GGTCTTGGTCCTTAGCCACT-3′), IL-10 (forward, 5′-GC TGGACAACATACTGCTAACC-3′; reverse, 5′-ATT TCCGATAAGGCTTGGCAA-3′), TNFα (forward, 5′-AACT AGTGGTGCCAGCCGAT-3′; reverse, 5′-CTTCACA GAGCAATGACTCC-3′), TGF-β (forward, 5′-CCACCTG CAAGACCATCGAC-3′; reverse, 5′-CTGGCGAGC CTTAGTTTGGAC-3′), G6pdx (forward, 5′-CACAGTGGACG ACATCCGAAA-3′; reverse, 5′-GCAGGGCATTCATGTG GCT-3′), Pgd (forward, 5′-AAGCTGACATTGCACTGATCG-3′; reverse, 5′-CGGCGGGGCTTCTTTAGTT-3′), H6pd (forward, 5′-AGTGGAGGACTATCAGACCCT-3′; reverse, 5′-GGCGGCACACTGAAGTAGAAG-3′), Tkt (forward, 5′-ATCACAGCCTCAAGTCAGTGG-3′; reverse, 5′-TTCA GGTCATCGGGTTGCAC-3′), Taldo1 (forward, 5′-GTGGG GCGCATCCTTGATT-3′; reverse, 5′-TGGTCTTGT AGCCGAACTTCT-3′), Aldoc (forward, 5′-TGTACCGCCAG GTCCTATTCA-3′; reverse, 5-GAGGCACTACACCCTTG TCAA-3′), and Pfkl (forward, 5′-GGAGGCGAGAACATCAA GCC-3′; reverse, 5′-GCACTGCCAATAATGGTGCC-3′). The miRNA levels were standardized to hsa-U6 (CD201-0145) as an internal control. The primer (Ribobio, Guangzhou, China) used to measure miRNA expression is listed here, mmu-miR-323-5p (CD202-0153).

### Histological Assessment

Histopathologic examination of the skin, lung, and kidney samples was performed as previously reported. Briefly, tissues were collected, fixed in 4% paraformaldehyde, dehydrated, paraffin-embedded, and sectioned for HE staining according to the manufacturer’s protocol. The kidney was scored by measuring glomeruli size in 5 fields per sample and then averaging groups. Skin and lung injury scores were quantified as previously described (19, 20). For immunofluorescent staining, paraffin-embedded tissue blocks were cut into 4-μm sections. Then, the sections were dewaxed and hydrated, and antigen retrieval was performed by boiling (in a 600-W microwave oven) in citrate buffer (2.1 g sodium citrate/L, pH 6) for 15 min. After the sections were cooled to room temperature, they were washed three times in PBS and then incubated with QuickBlock™ Blocking Buffer for Immunol Staining (Beyotime, Jiangsu, China) for 15 min to block the non-specific binding of immunoglobulins. After that, the sections were incubated with primary antibody at 4°C overnight. The following antibodies were used: C3 (Abcam, Cambridge, MA, USA) and goat anti-mouse IgG antibody (Abcam, Cambridge, MA, USA). After washing, the sections were incubated with corresponding secondary antibodies for 60 min. The percentages of positive immunofluorescent staining areas of IgG or C3 were measured.

### Statistical Analysis

Data from at least three independent experiments, were analyzed with a statistical software package (GraphPad Prism 9) and were presented as the means ± SEM. All data were analyzed by the two-tailed unpaired Student’s *t*-test. For all statistical analyses, statistical significance is indicated by a single asterisk (*p*-value < 0.05), two asterisks (*p*-value < 0.01), three asterisks (*p*-value < 0.001), and four asterisks (*p*-value < 0.0001).

## Results

### ACs Inhibit Macrophage PPP Activity *In Vitro* and *In Vivo*

**Figure 1:**
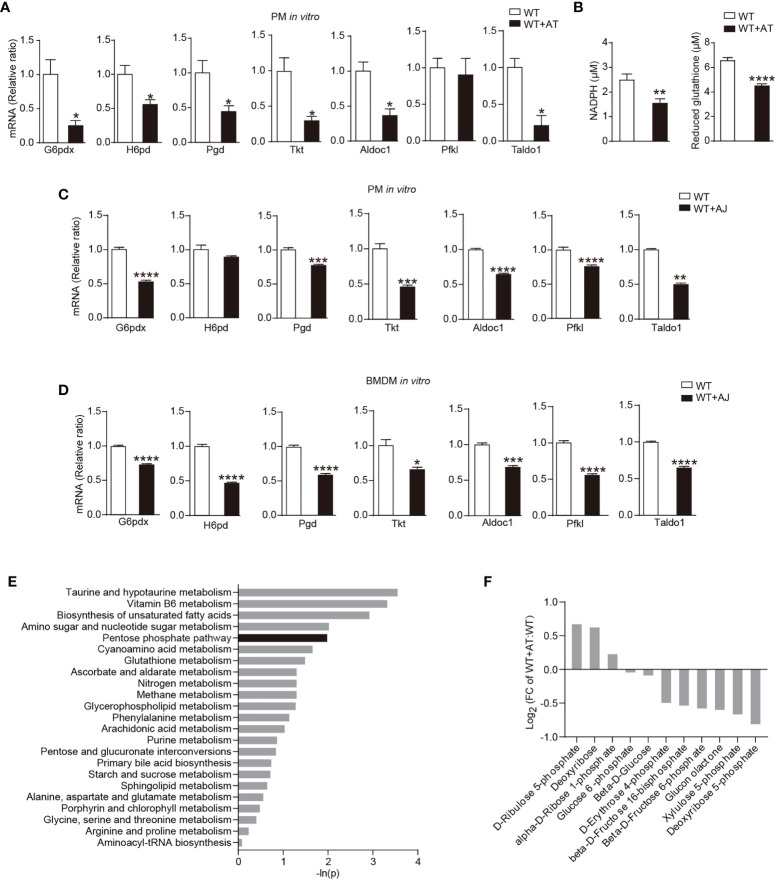
ACs inhibit macrophage PPP activity. **(A, B)** Thioglycolate-elicited peritoneal macrophages (PMs) from WT mice were incubated with or without apoptotic thymocytes for 24 h *in vitro*. **(A)** Changes of gene expression in PPP by quantitative RT-PCR (*n* = 3). **(B)** Levels of NADPH and reduced glutathione in macrophages (*n* = 6). **(C, D)** Thioglycolate-elicited peritoneal macrophages (PMs) **(C)** or bone marrow-derived macrophages (BMDMs) **(D)** from WT mice were co-incubated with apoptotic human Jurkat T cells for 24 h; mRNA expression of PPP-related genes in macrophages was measured by quantitative RT-PCR (*n* = 3). **(E, F)** Thioglycolate-elicited peritoneal macrophages from WT mice were incubated with or without apoptotic human Jurkat T cells for 6 h *in vitro*, high-throughput metabolomics was performed (*n* = 6), and enrichment results of metabolomic pathways **(E)** and the relative content of metabolites in PPP **(F)** were shown (Negative ion model). Results were expressed as mean ± SEM. *p < 0.05, **p < 0.01, ***p < 0.001, and ****p < 0.0001 (two-tailed Student’s *t*-test).

To explore whether PPP contributes to the macrophage adaptation to the ACs removal, we first detected whether ACs altered macrophage PPP activity *in vitro*. Levels of several PPP-related genes, including G6pdx (**Figure 1A**), NADPH, and reduced glutathione (**Figure 1B**) were reduced in peritoneal macrophages following ACs incubation, indicating that the engulfment of ACs greatly reduced the activity of PPP in macrophages. To exclude the potential contamination of AC-derived PPP-associated genes to the signals detected in peritoneal macrophages, the apoptotic human Jurkat cells were used, which inhibited the mRNA levels of PPP-related genes in peritoneal macrophages as well (**Figure 1C**). Furthermore, apoptotic Jurkat cells also decreased mRNA levels of PPP-related genes in bone marrow-derived macrophages (BMDMs) (**Figure 1D**).

Furthermore, we performed unbiased liquid chromatography-tandem mass spectrometry (LC-MS/MS) to directly measure PPP-related metabolic remodeling during efferocytosis. Unbiased analysis of the LC-MS/MS readings identified reproducible global changes in metabolites (**Figures S1A, B**). Over 74 biochemicals changed significantly, of which 44 increased and 30 decreased (**Figure S1C**). Metabolite set enrichment analysis highlighted noteworthy decrease in PPP metabolism (**Figure 1E**). PPP metabolites, such as deoxyribose 5-phosphate and xylulose 5-phosphate, were significantly decreased following AC phagocytosis (**Figure 1F**). Notably, while levels of total glutathione, including reduced glutathione and oxidized glutathione, were significantly increased (**Figure S1C**), concentrations of reduced glutathione were greatly reduced (**Figure 1B**), that is, in line with the function of PPP in providing NADPH and generating reduced glutathione.

Collectively, data here showed that ACs inhibited macrophage PPP activity.

### PPP Regulates Immune-Silent Clearance of ACs by Macrophages *In Vitro* and *In Vivo*

**Figure 2:**
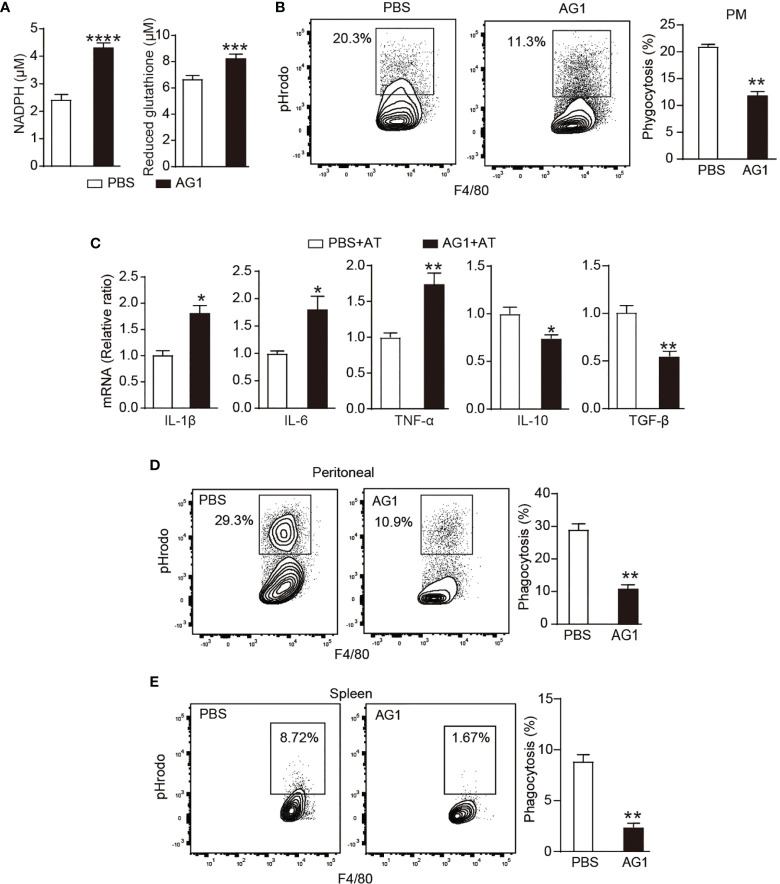
PPP regulates immune-silent clearance of ACs by macrophages *in vitro* and *in vivo*. **(A–C)** Cultured thioglycolate-elicited peritoneal macrophages from WT mice were pre-treated with AG1 (3 μM) or PBS *in vitro* for 24 h. **(A)** Levels of NADPH and reduced glutathione in macrophages were detected (*n* = 6). **(B)** *In vitro* phagocytosis of pHrodo-labeled apoptotic thymocytes was measured by flow cytometry (*n* = 3). **(C)** PMs were incubated with apoptotic thymocytes for 24 h, mRNA levels of inflammatory cytokines in macrophages were measured by quantitative RT-PCR (*n* = 3). **(D, E)** WT mice were given with AG1 (10 mg/kg/day, i.p.) or PBS for 24 h; pHrodo-labeled apoptotic thymocytes were i.p. **(D)** or i.v. **(E)** injected to mice; after indicated times, engulfment of apoptotic thymocytes by F4/80^+^ peritoneal **(D)** or spleen macrophages **(E)** was detected by flow cytometry (*n* = 3). Results were expressed as mean ± SEM. *p < 0.05, **p < 0.01, ***p < 0.001, and ****p < 0.0001 (two-tailed Student’s *t*-test).

To explore the contribution of PPP activity to efferocytosis, we used AG1 that can increase the activity of G6PD (21) to correct the efferocytosis-reduced PPP activity in macrophages. During efferocytosis, AG1 treatment increased concentrations of NADPH and reduced glutathione in macrophages *in vitro* (**Figure 2A**), indicating the effectiveness of AG1 to restore the activity of PPP. Meanwhile, AG1 treatment induced a ~50% reduction of AC phagocytosis *in vitro* (**Figure 2B**), suggesting an important contribution of PPP activity to efferocytosis. In addition, AG1 also greatly induced the expression of inflammatory cytokines TNF-α, IL-1β, and IL-6 but reduced the production of immunosuppressive mediators TGF-β and IL-10 during efferocytosis (**Figure 2C**).

The altered inflammatory response following AG1 treatment during efferocytosis may be due to less phagocytosis, leading to apoptotic cell accumulation and secondary necrosis, resulting in unregulated inflammatory response or impaired tolerogenic response during efferocytosis. To distinguish these two different mechanisms, we discarded apoptotic cells following the incubation of macrophages with apoptotic cells for 4 h, and macrophages were cultured for 6 h more before they were used for the measurement of inflammatory cytokines. Results showed that the treatment of AG1 also increased the mRNA levels of TNF-α and IL-1β but reduced IL-10 and TGF-β during efferocytosis, suggesting that the activation of the PPP activity regulates the intrinsic tolerogenic pathway during apoptotic clearance (**Figure S2A**).

In addition, accumulated data have shown that peritoneal cavity macrophages are functionally and phenotypically heterogeneous, which mainly include monocyte-derived inflammatory macrophages and tissue resident macrophages (22, 23). Since peritoneal macrophages used in our above experiments were thioglycolate-elicited macrophages, which are monocyte-derived inflammatory macrophages, we further evaluated the effect of PPP on peritoneal resident macrophages. We isolated the peritoneal macrophages without pre-thioglycolate administration. While AG1 treatment significantly induced IL-1β levels, expressions of other cytokines, including IL-6, TNF-α, TGF-β, and IL-10, were not significantly influenced in resident peritoneal macrophages (**Figure S2B**). This effect was different from that observed in inflammatory macrophages, indicating different contributions of PPP activity to regulating tolerogenic pathway during apoptotic clearance in resident macrophages and inflammatory macrophages.

We further investigated the involvement of PPP in efferocytosis *in vivo*. WT mice were given AG1 for 1 day and then applied for *in vivo* efferocytosis assay as described above. AG1 greatly reduced the AC engulfment by F4/80+ peritoneal macrophages (**Figure 2D**) and spleen macrophages (**Figure 2E**).

We also applied 6AN or DHEA to inhibit PPP activity. However, these PPP inhibitors did not show significant effect in promoting macrophage phagocytosis (**Figure S2C**), which might be due to the reduction of PPP activity after ACs phagocytosis, so PPP inhibitors could not further reduce PPP activity.

### ACs Regulate the PPP Activity Through miR-323-5p

**Figure 3:**
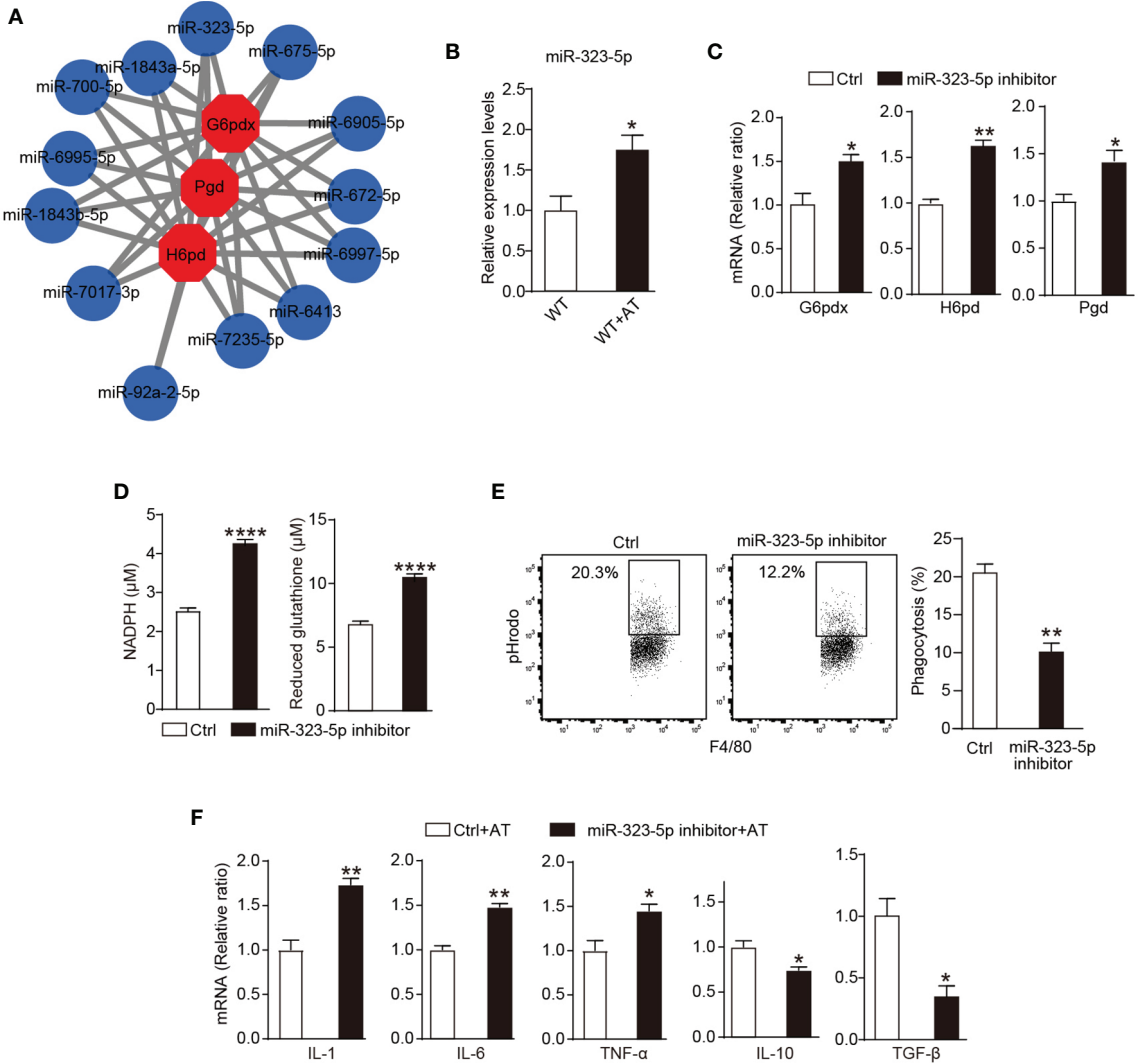
ACs regulate the PPP activity through miR-323-5p. **(A)** Top 13 of G6pdx, Pgd, and H6pd binding miRNAs were predicated by miRWalk database. **(B)** Thioglycolate-elicited peritoneal macrophages were incubated with or without apoptotic thymocytes for 24 h, and expression of miR-323-5p was assessed by quantitative RT-PCR (*n* = 3). **(C–F)** *In vitro* cultured WT mice thioglycolate-elicited peritoneal macrophages were treated with or without miR-323-5p inhibitor (100 nM) for 24 h. **(C)** mRNA levels of G6pdx, H6pd, and Pgd of macrophages were measured by quantitative RT-PCR (*n* = 3). **(D)** Concentrations of NADPH and glutathione in macrophages (*n* = 6). **(E)** *In vitro* phagocytosis of apoptotic thymocytes by macrophages was detected by flow cytometry (*n* = 3). **(F)** Thioglycolate-elicited peritoneal macrophages were incubated with apoptotic thymocytes for 24 h, and mRNA levels of inflammatory cytokines in macrophages were measured by quantitative RT-PCR (*n* = 3). Results were expressed as mean ± SEM. *p < 0.05, **p < 0.01, and ****p < 0.0001 (two-tailed Student’s *t*-test).

Our previous investigation revealed that ACs regulated macrophage activity through Dicer (15). Given the essential role of Dicer in miRNA genesis, we subsequently explored miRNAs that are responsible for the PPP activity in macrophages during efferocytosis. Several miRNAs, such as miR-1a-3p (24), -24-3p (25), and -206-3p (26), have been reported to downregulate G6PDX expression, which is the rate-limited enzyme of PPP. Our investigations focused on miRNAs that regulate PPP activity. Considering that each miRNA may control ~200 genes, and conversely, one mRNA transcript may be targeted by multiple miRNAs (27), we tried to identify miRNAs that regulate the expression of more members of PPP and therefore may play more important roles in modulating PPP activity. The online open database miRWalk (28) was applied to predict miRNAs that can bind to different members of PPP. The top 13 predicted miRNAs are shown in **Figure 3A**, among which miRNA-323-5p ranked number one by TargetScan (29) (**Figure S3A**). MiRNA-323-5p has only been reported to target insulin-like growth factor 1 receptor to promote cell apoptosis (30), so we investigated whether miRNA-323-5p regulated PPP activity and efferocytosis in macrophages.

The addition of ACs increased levels of macrophage miR-323-5p (**Figure 3B**). We further investigated the ability of miR-323-5p to modulate PPP activity and dying cell removal. In peritoneal macrophages, miR-323-5p inhibitor greatly increased the mRNA levels of PPP member G6pdx, H6pd and Pgd (**Figure 3C**), and concentrations of NADPH and reduced glutathione (**Figure 3D**). Furthermore, miR-323-5p inhibitor reduced the macrophage phagocytosis of ACs by ~50% (**Figure 3E**). Additionally, the miR-323-5p inhibitor also greatly induced the production of inflammatory cytokine TNF-α, IL-1β, and IL-6 but reduced the production of immunosuppressive mediators TGF-β and IL-10 during efferocytosis (**Figure 3F**). Together, these data suggested that miR-323-5p was essential in regulating PPP activity and AC clearance in macrophages.

### Interfering the PPP Activity Regulates the Process of SLE

**Figure 4:**
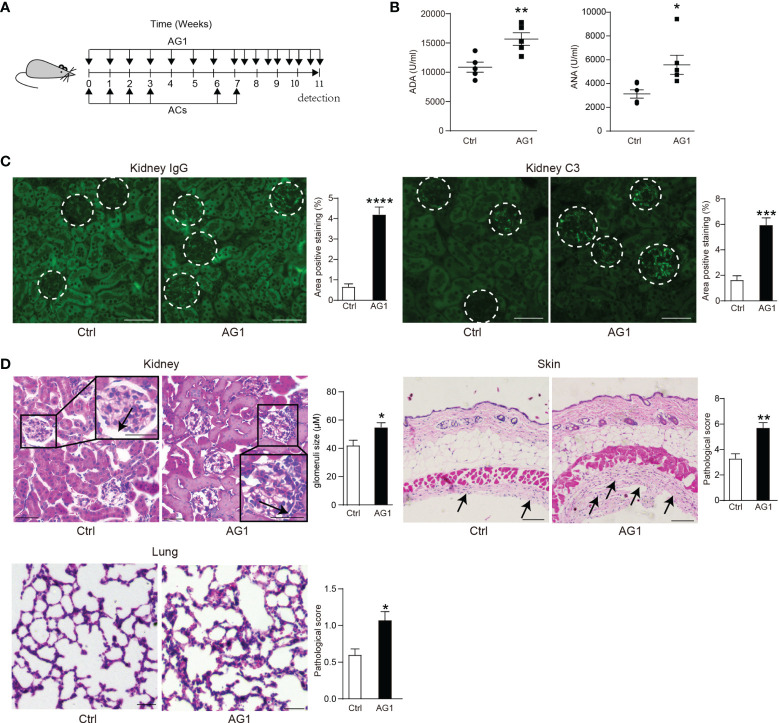
Interfering the PPP activity regulates the process of SLE. **(A)** Eight-week-old female WT mice were intravenously injected with 1.5 × 10^7^ apoptotic thymocytes by four consecutive injections once a week; after 15 days, the injections were repeated twice. Meanwhile, 24 h before apoptotic cell injection, AG1(10 mg/kg, i.p.) was administered weekly (AG1 is still injected at a fixed time during the 15-day break). After the last apoptotic cells injection, AG1 was injected twice per week. The same volume of PBS was injected into the control group. **(B)** Serum concentrations of anti-dsDNA antibodies (ADA) (*n* = 5) and anti-nuclear antibodies (ANA) (*n* = 6) were detected by ELISA. **(C)** Representative images and percentages of positive staining areas of IgG and C3 in kidneys of AG1-treated SLE mice and untreated SLE mice (*n* = 5, bar = 50 μm). **(D)** Representative images and quantitative assessment of hematoxylin-and-eosin (HE) staining of the kidney (bar = 50 μm), skin (bar = 100 μm), and lungs (bar = 100 μm) from AG1-treated SLE mice and untreated SLE mice (*n* = 5 per group). *p < 0.05, **p < 0.01, ***p < 0.001, and ****p < 0.0001 (two-tailed Student’s *t*-test).

We induced a mouse SLE model through tail vein injection of apoptotic thymocytes over an 8-week period from 8 weeks of age. We applied G6PD activator AG1 to enhance PPP; untreated ACs-induced SLE mice served as control (**Figure 4A**). Considering that anti-nuclear antibodies (ANA) and anti-dsDNA antibodies (ADA) in serum are the prominent markers of SLE, we first measured levels of serum ADA and ANA in AG1- or control-SLE mice. Compared with control, AG1 increased the serum level of ADA and ANA (**Figure 4B**).

The deposition of autoantibodies as immune complexes in the kidney is another hallmark of SLE. In AG1-treated mice, more prominent accumulation of IgG and complement C3 in glomerulus was observed than in control mice (**Figure 4C**). Moreover, AG1 treatment increased glomerular size in SLE mice compared to their untreated counterparts (**Figure 4D**). In addition, more prominent inflammatory cell accumulation was observed in the skin and lung of AG1-treated SLE mice (**Figure 4D**).

Briefly, these data demonstrated that the enhancement of PPP activity can deteriorate the development of SLE-like disease.

## Discussion

Given apoptosis is the dominant modality of homeostatic cell turnover (2), novel pathways involved in regulating immunologically non-inflammatory dying cell clearance are of wide interest. Accumulated evidence indicated that engulfment of dying cells enhances the phagocytic ability of phagocytes to adapt to the changing microenvironment (6, 7). However, pathways that modulate the tolerogenic phagocytosis remain indistinct. Our work here uncovers that PPP is an important pathway to orchestrate efferocytosis in macrophages. We found that ACs inhibited macrophage PPP activity and the activation of PPP greatly reduced ACs engulfment and increased inflammatory cytokine production in macrophages. This result supported that PPP played an essential role in immune-tolerant dying cell removal and the maintenance of immune tolerance. Meanwhile, miRNA-323-5p regulates the PPP activity and immune-silent AC uptake in macrophages. miRNA-323-5p inhibitor increased PPP activity, reduced the phagocytosis of ACs, and induced an inflammatory response during efferocytosis in macrophages. Accordingly, enhancement of PPP promoted impaired efferocytosis that contributed to the progress of lupus-like symptoms. These observations show that ACs phagocytosis inhibits PPP to orchestrate tolerogenic dying clearance and interfering with the PPP can also harness immune-silent dying cell clearance.

Although accumulated lines of evidence have established an important interplay between efferocytosis and cellular metabolic changing (6–11), the involvement of PPP in efferocytosis remains scarce. Here, we found that PPP activity and metabolites were greatly decreased during efferocytosis, and increased PPP activity was related with reduced AC engulfment and inflammatory response during efferocytosis. The PPP is a parallel metabolic pathway of glycolysis, which generates ribose-5-phosphate to produce nucleotide synthesis and NADPH to produce fatty acid or reduced glutathione (12). An increase in PPP activity is observed in LPS-stimulated macrophages, and a defective PPP reduces inflammatory macrophage responses (31, 32), which is in line with our observation that increased PPP activity induced inflammatory response during efferocytosis. While our observations clearly show that alteration of the PPP contributes directly to efferocytosis, a better understanding of mechanisms underlying its contributions to efferocytosis will be valuable in developing therapeutic strategies targeting impaired efferocytosis and related diseases.

miRNAs are essential in many important biological processes. Recent studies have determined a link between miRNAs and efferocytosis (33). For example, miRNA-126 (34) and miRNA-21 (35) were found to promote efferocytosis in macrophages, but miRNA-34a negatively regulates efferocytosis (36). Dysregulation of miRNA may lead to various pathological conditions associated with defective efferocytosis, such as atherosclerosis. Notably, each miRNA may control about 200 genes; conversely, one mRNA transcript can be targeted by multiple miRNAs (27). However, the global changes of miRNAs following efferocytosis remain unknown. Our data showed that inhibiting miRNA-323-5p results in improved PPP activity and impaired efferocytosis.

SLE is a typical systemic autoimmune disease, which affects various organs. Currently, there is no optimal treatment (37). Growing studies have reported that macrophages play a significant role in SLE, involving high inflammatory cytokine production and defective efferocytosis (38). Generally, ACs clearance by phagocytes is rapid and non-inflammatory to avoid secondary necrosis. Low phagocytic function results in abnormal accumulation of corpse, facilitating the development of SLE (39). We used activator of PPP to intervene in this pathological process and found that levels of ANA and ADA were increased compared with control, and inflammatory cell infiltration in kidney was more obvious, indicating deteriorating SLE development. While our data manifest that inhibiting the elimination of dead cells through promoting PPP activity is related to the progress of SLE in mice, further investigation is needed to explore the involvement of PPP in the development of human SLE.

## Data Availability Statement

The original contributions presented in the study are included in the article/**Supplementary Material**. Further inquiries can be directed to the corresponding authors.

## Ethics Statement

The animal study was reviewed and approved by Laboratory Animal Welfare and Ethics Committee of the Army Medical University.

## Author Contributions

ZZ provided the idea and conceived and designed the experiments. DH, JJ, and QM performed the experiments. ZW, YL, and TL provided the technical support. BL and ZZ analyzed and interpreted the data. ZZ, BL, and DH wrote the draft of the manuscript. ZZ revised the manuscript. ZZ and BL supervised the study. All authors contributed to the article and approved the submitted version.

## Funding

This work was supported by grants (81671559 and 82071778) from the National Natural Science Foundation of China (ZZ).

## Conflict of Interest

The authors declare that the research was conducted in the absence of any commercial or financial relationships that could be construed as a potential conflict of interest.

## Publisher’s Note

All claims expressed in this article are solely those of the authors and do not necessarily represent those of their affiliated organizations, or those of the publisher, the editors and the reviewers. Any product that may be evaluated in this article, or claim that may be made by its manufacturer, is not guaranteed or endorsed by the publisher.
