# Supplementary material for: Pentose Phosphate Pathway Regulates Tolerogenic Apoptotic Cell Clearance and Immune Tolerance
Source: Front Immunol. 2022 Jan 10;12:797091. doi: 10.3389/fimmu.2021.797091 (PMC8784392; doi:10.3389/fimmu.2021.797091)
Supplement: Supplementary file 1 [file DataSheet_1.docx]

Supplementary Material


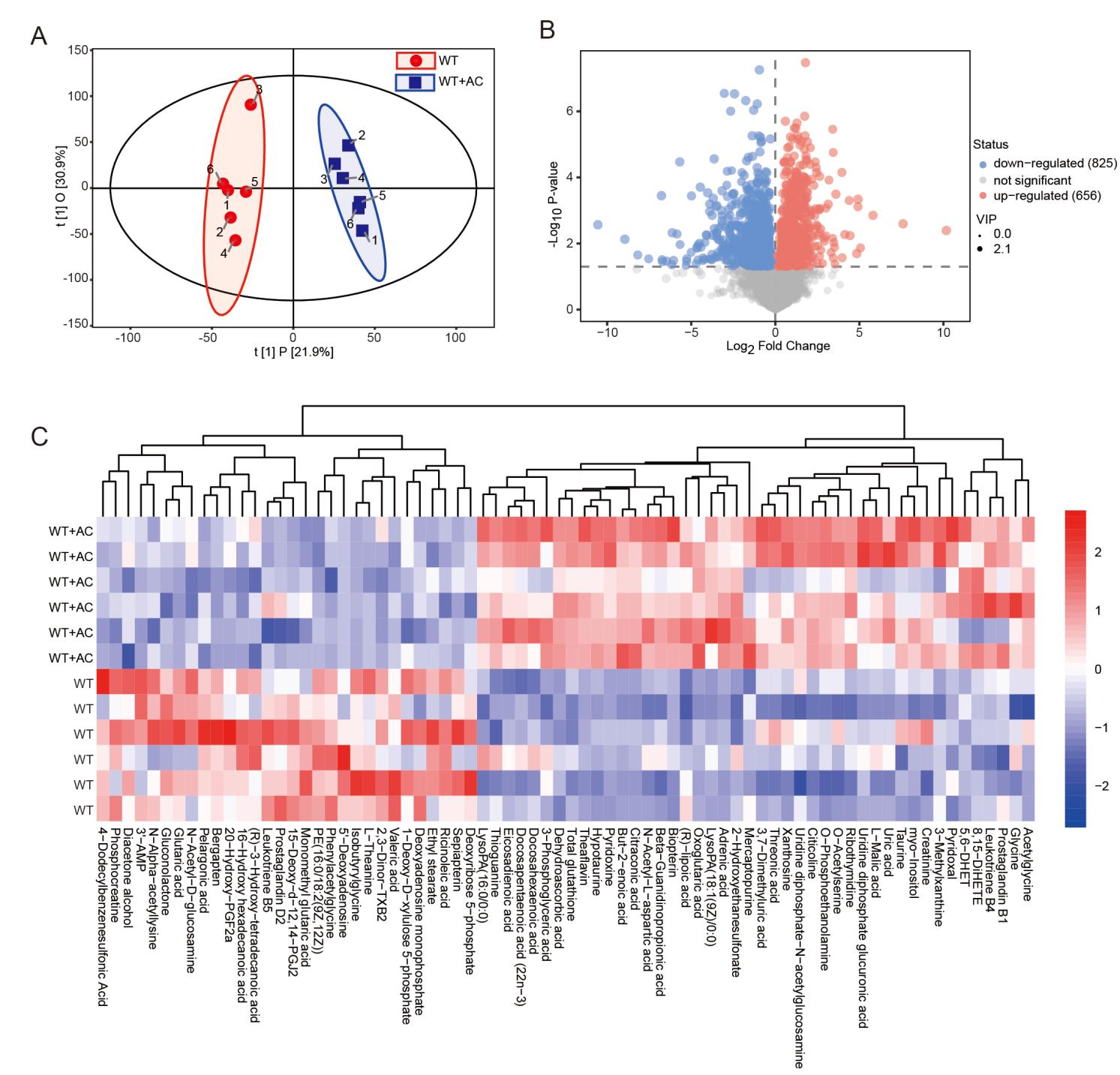


**Supplementary Figure 1.** For **A-C:** Unbiased liquid chromatography-tandem mass spectrometry (LC-MS/MS). **A-B**: Unbiased analysis of the LC-MS/MS readings identified reproducible global changes in metabolites. **A**: *OPLS-DA* score plot of metabolite profiles. **B**: Volcano plot of metabolite profiles. **C**: Clustering heat map of differential metabolites, over 74 total biochemicals were altered with a p < 0.05, with 44 induced and 30 downregulated.


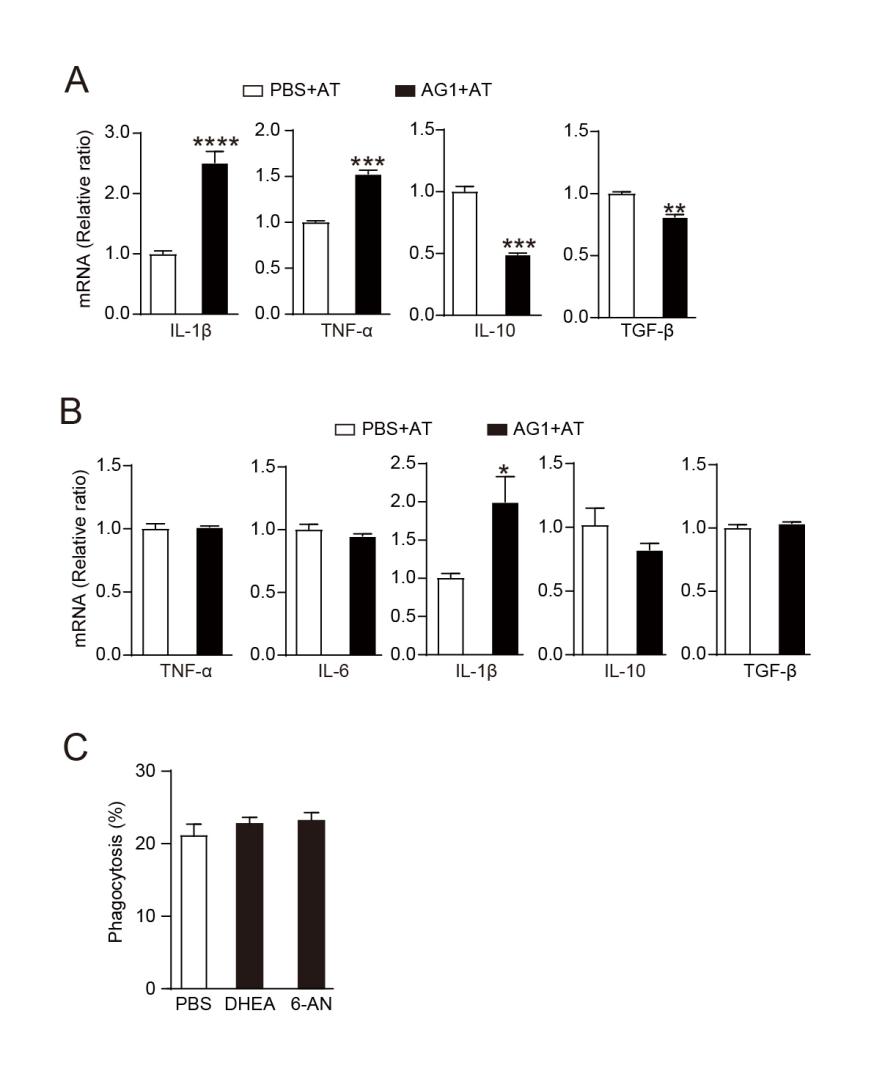


**Supplementary Figure 2**. For **A-C**: PMs were pre-treated with AG1 (3μM) or PBS *in vitro* for 24 hours. **A**: Thioglycolate-elicited peritoneal macrophages were incubated with apoptotic thymocytes for 4 hours, then cultured for 6 hours more，mRNA levels of inflammatory cytokines in macrophages were measured by quantitative RT-PCR (n = 3). **B**: Peritoneal resident macrophages were incubated with apoptotic thymocytes for 6 hours, the expression of inflammatory cytokines in macrophages were measured by quantitative RT-PCR (n = 3). **C**: Thioglycolate-elicited peritoneal macrophages macrophages were treated with DHEA or 6AN for 24 hours, *in vitro* phagocytosis of pHrodo-labelled apoptotic thymocytes was measured by flow cytometry (n = 3). Results were expressed as mean ± SEM. *P < 0.05, **P < 0.01, ***P < 0.001 and ****P < 0.0001 (two-tailed Student’s t-test).


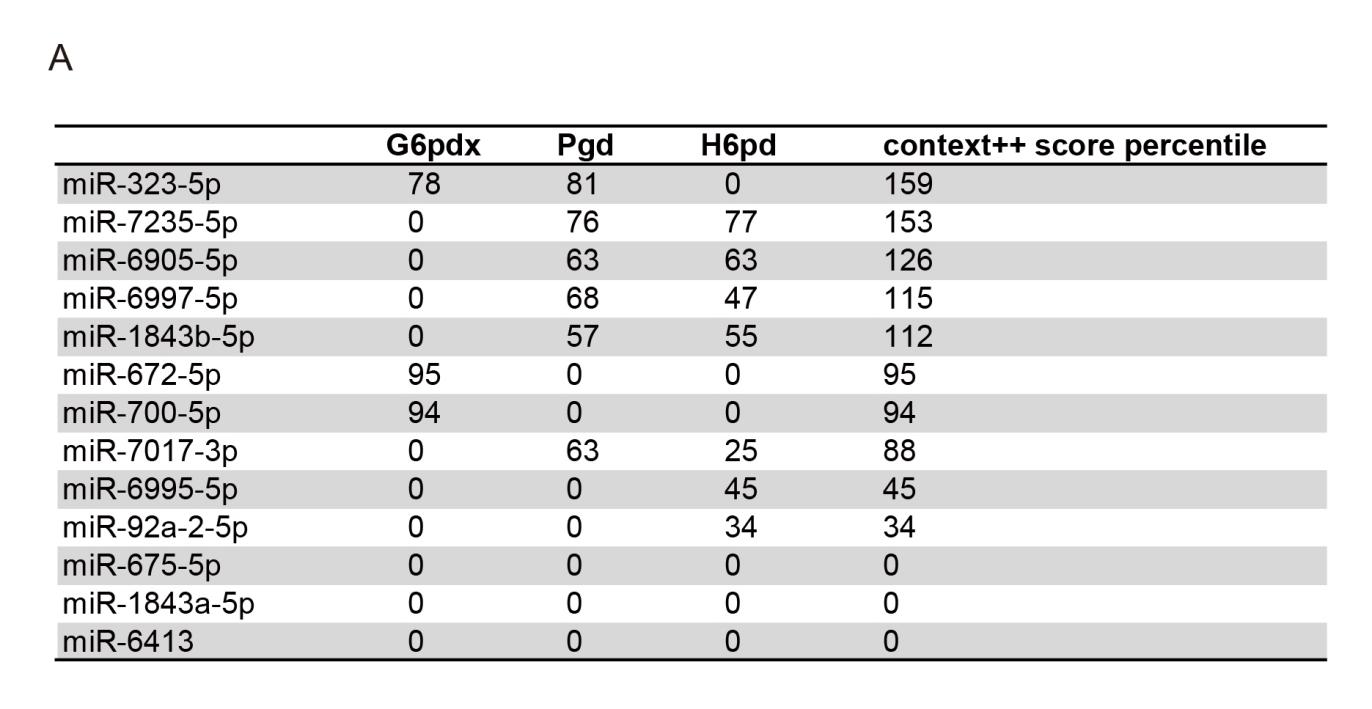


**Supplementary Figure 3.** **A:** The top miRNAs were predicted by TargetScan.
